# Driving Mechanism Model for the Supply Chain Work Safety Management Behavior of Core Enterprises—An Exploratory Research Based on Grounded Theory

**DOI:** 10.3389/fpsyg.2021.807370

**Published:** 2022-02-03

**Authors:** Qiaomei Zhou, Qiang Mei, Suxia Liu, Jingjing Zhang, Qiwei Wang

**Affiliations:** ^1^School of Management, Jiangsu University, Zhenjiang, China; ^2^College of Economic and Management, Zhengzhou University of Light Industry, Zhengzhou, China

**Keywords:** core enterprise, work safety management behavior in supply chain, driving factors, influence path, guiding policy, grounded theory

## Abstract

Guiding core enterprises to participate in supply chain work safety governance is an innovative mode of work safety control, which has an important impact on improving the work safety level of small and medium-sized enterprises in the supply chain. Through in-depth interviews, the grounded theory is adopted to explore the driving factors of work safety management behaviors of core enterprise. It is found that the work safety management behavior of the core enterprise is driven by both internal and external factors. External driving factors are the main category of institutional pressure composed of regulatory pressure, normative pressure, and cognitive pressure. Internal driving factors are divided into the main category of behavioral awareness and the behavioral capability of the core enterprises. The behavioral awareness is composed of responsibility sense, problem cognition and behavioral effect perception. The behavioral capability is composed of safety management strength and safety coordination capability. Institutional pressure, behavioral awareness, and behavioral capability all influence the work safety management behavior of core enterprise in supply chain significantly, but with different ways and paths. On this basis, the driving mechanism model of the above three main categories on the work safety management behavior in core enterprise supply chain is explored. The research can provide targeted policy ideas and implementation paths for the government to formulate effective guiding policies to promote the work safety management behavior of core enterprise.

## Introduction

There are serious supply chain work safety problems because of a large number of small and medium-sized manufacturing suppliers in the supply chain. In China, due to the backward technology and equipment, incomplete safety facilities and equipment, shortage of funds for work safety and chaotic safety management in most small and medium-sized manufacturing enterprises, the level of work safety is low and there are more serious occupational hazards and safety accidents. On the one hand, small and medium-sized manufacturing enterprises are suspended and shut down for work safety issues, which directly affects the supply of core enterprises in the supply chain. For example, in September 2017, the auto parts manufacturer, Schaeffler Greater, was severely affected after Shanghai Jielong, which produced needle rollers, was ordered to stop production. On the other hand, the occurrence of occupational hazards or safety accidents in small and medium-sized manufacturing enterprises has a serious negative impact on the reputation and corporate value of core enterprises in the supply chain. For example, in 2009 employees of Apple’s supplier were poisoned with n-alkane, as a result, Apple was criticized by environmental organizations and its reputation was damaged. In order to prevent the risk of supply chain safety accidents and occupational disease injuries, core enterprises need to adopt work safety management behaviors for their suppliers. Work safety management behavior in supply chain refers to the behavior that core enterprises to pay attention to the work safety of suppliers in the process of cooperation with them, and take corresponding measures to improve the safety management level of suppliers, prevent safety accidents and reduce occupational injury incidents (Ustailieva et al., 2012). According to Seuring and Müller (2008), compared with small and medium-sized manufacturing enterprises, core enterprises are in the leading position of the supply chain, with more advanced work safety concepts, technologies and management tools, so they might have the ability and obligation to take more social responsibility to improve the safety performance of their suppliers. At the same time, due to the order-dependent nature of small and medium-sized manufacturing enterprises, they would value the safety standard requirements and safety management of the core enterprises, and then adopt active work safety behavior.

The government and all sectors of society have gradually recognized the importance of work safety management behaviors in supply chain (Peckham et al., 2017; Zhou et al., 2020). In practice, some famous brand companies have made some useful attempts on work safety management behaviors in supply chain in China, such as multinational companies like Volkswagen, IKEA, Covestro, etc., and the local Chinese company, Huawei. However, most other companies are reluctant to participate in or do not know how to carry out work safety management in supply chain, i.e., there is not enough motivation for work safety management in supply chain. In traditional supply chain management, profit maximization is the goal of all enterprises. Although work safety management in supply chain can improve the safety level of the whole supply chain, it increases the operation cost of enterprises (Walters and James, 2011). On the one hand, there are few enterprises taking the initiative to extend their corporate social responsibility to carry out work safety management in supply chain, for example, they pay little attention to the work safety level of upstream and downstream enterprises in the supply chain and do not know enough about the benefits of safety management in supply chain. Moreover, the supplier chain of core enterprises is long and complex, so it is difficult to implement work safety management in supply chain (Alsamawi et al., 2017). On the other hand, the higher requirements of the core enterprises on the suppliers in terms of price, quality, and delivery of goods or services indirectly lead to heavier and more intensive workloads in the suppliers, especially small and medium-sized enterprises, which subsequently lead to adverse health and safety conditions for the workers (Bhattacharya and Tang, 2013; Siganporia et al., 2016). Therefore, strengthening the guidance of supply chain management for core enterprises and motivating them to adopt work safety management in supply chain has become an urgent research topic.

In order to encourage more core enterprises to adopt work safety management behaviors in supply chain, it is necessary to clarify the deep-seated factors driving core enterprises’ work safety management in supply chain and their influencing paths on management behaviors. Therefore, this paper analyzes the motives of core enterprises to participate in safety management in supply chain from a behavioral perspective by using in-depth interviews and grounded theory, that is, the internal and external influencing factors of core enterprises’ work safety management behaviors in supply chain and their mechanisms on enterprise management behavior decisions are explored in depth from a microscopic perspective. As a scientific and qualitative research method, grounded theory is suitable for studying the “how” and “why” questions. In this paper, open-ended questions are set up to discuss with supply chain managers the specific safety management behaviors in supply chain and the reasons for the management behaviors. Then, according to the interview data, the influencing factors of work safety management behavior in supply chain and the conduction relationship between influencing factors and management behavior are gradually extracted. This paper is an important supplement to work safety management in supply chain, and it has great policy guidance significance to guide more core enterprises to implement safety management in supply chain effectively.

## Literature Review

Currently, there is little literature focusing on supply chain work safety management, but work safety of enterprises in social sustainability is the focus of social attention, and work safety element is also an important part of the sustainable development indicator of enterprises (Tsuda and Takaoka, 2006). Therefore, reviewing the literature on the drivers of supply chain sustainability management can provide a theoretical basis for the subsequent identification of the drivers of supply chain work safety management behavior in this paper. Supply chain sustainability management is essential for corporate to achieve sustainable competitive advantages (Dai et al., 2021). Current research in supply chain sustainability management focuses on environmental activities (Shibin et al., 2017; Boiral et al., 2019; Siems et al., 2021). However, it is shifting to focus on social requirements, because the social performance of the supply chain has a significant impact on stakeholders and supply chain performance (Mani et al., 2020). This paper will provide an overview of the drivers of supply chain sustainability management from both external and internal perspectives.

### The External Factors

Sustainable management in supply chain mainly comes from the adjustment and shaping of external pressure, including institutional pressure, stakeholder pressure and possible sustainable risks. The institutional theory and stakeholder theory are involved. In 2011, Lee proposed a theoretical framework that combined institutional theory and stakeholder theory to explain how enterprises choose social responsibility strategies. It proposed that stakeholders obtain legitimacy and power from the institution. Institutional pressure influences the social behavior of enterprises through the stakeholder mechanism. The two are interdependent to form a specific external influence structure, which shapes how enterprises construct social responsibility strategies (Lee, 2011). Institutional theory studies different pressures and their impact on enterprise management decisions. Institutional pressure [including regulatory pressure, normative pressure and cognitive pressure (Scott, 1995)] is regarded as an important driving factor for enterprise supply chain management (Srivastava et al., 2021), because any enterprise must contend with institutional factors in management (Zeng et al., 2017). For example, government policies, laws and regulations may have a positive impact on sustainable supply chain management (Zhu et al., 2005). When the product raw material country lacks a policy and legal framework to manage the sustainability issues in complex supply chain, transnational corporations will evade supply chain governance (Boström and Micheletti, 2016). In addition, regulatory documents issued by non-governmental organizations can also encourage enterprises to fulfill social responsibilities (Phan and Baird, 2015). The external institutional environment not only shapes and strengthens the guiding principles of the organization, but also ensures that the organization abides by external rules, norms and values. Organizations respond to institutional pressure in different ways, such as compromise, avoidance, provocation or utilization (Oliver, 1991). In general, industries that are more regulated or its products and services directly affect people’s lives, such as the food industry, pharmaceutical industry and automobile industry, will face higher institutional pressures. However, some studies show that it is not the industry but the specific behaviors within the industry that need to match higher institutional pressures, such as: child labor, pollution, unsustainable production methods, etc. (Srivastava et al., 2021). In turn, institutional pressure has promoted closer cooperation between buyers and sellers, such as developing green supply chain management to smoothly meet the requirements of mandatory regulations (Jazairy and von Haartman, 2020).

Stakeholders can mobilize public opinion to support or oppose the sustainability performance of the organization. From the perspective of resource dependence, stakeholders influence organizational behavior by influencing the acquisition of key resources, that is, they manipulate the flow of resources to the organization (Frooman, 1999). Sustainability has transcended the boundaries of the organization, so stakeholders put pressure on the sustainable development of the entire supply chain (Cantele and Zardini, 2020). Since larger enterprises are more likely to be scrutinized by stakeholders, they are more willing to transfer some of the pressure to supply chain partners (Parmigiani et al., 2011). Assistance from non-governmental organizations can help core enterprises improve their supply chain sustainable management capabilities (Stekelorum et al., 2020). With the help of third-party organizations, enterprises can obtain resources that cannot be obtained independently (Crespin-Mazet and Dontenwill, 2012) and establish social networks, that is, strategic bridging (Stafford et al., 2000). According to the relational theory (Dyer and Singh, 1998), enterprises operate in a relationship network, enabling them to create value that they cannot create independently (Lavie, 2006). In fact, the relationship among organizations will lead to the development of new resources, and new resources will become a competitive advantage (Garcia-Perez-de-Lema et al., 2017). The motivation of sustainable management of the enterprise supply chain is also affected by customers (Kot, 2018; Mani et al., 2020), such as pressure and support from customers, including technical support (Hoogendoorn et al., 2015), knowledge improvement (Touboulic and Walker, 2015), and enhancement of its legitimacy (El Baz et al., 2016).

Some scholars put forward the sustainable risk, that is, failure to comply with stakeholders’ sustainability requirements may cause sustainability risks, including consumer boycotts, reputation damage, labor disputes, economic losses or legal proceedings, further damaging the financial performance of the enterprise and its supply chain (Hossan Chowdhury and Quaddus, 2021). For example, brand such as Nike, Adidas, Disney, and C&A are facing consumer resistance and close scrutiny by stakeholders due to the disclosure of sweatshop workers in the upstream supply chain (Busse et al., 2016). In some supply chain members, good sustainability practices may be ineffective due to poor sustainability practices of other supply chain members (which causes risks). Because of the knock-on risk, the core enterprise should be responsible for the behavior of the decentralized supply chain enterprises (Van Tulder et al., 2009; Wilhelm et al., 2016). Lacking proper sustainability governance may lead to poor sustainability practices among supply chain partners, bring risks to the entire supply chain, damage the overall reputation of the organization supply chain, and reduce performance (Faruk et al., 2001). In the supply chain, two concepts should be noted: interruption and vulnerability. Supply chain interruption risk = f (interruption, vulnerability) (Hossan Chowdhury and Quaddus, 2021), which is also an important driving factor for core enterprises to implement sustainable supply chain management. Seuring and Müller (2008) analyzed 191 papers on Sustainable Supply Chain Management (SSCM) published from 1994 to 2007, and summarized the following trigger factors of SSCM: legal requirements, customer requirements, maintaining competitive advantage, environmental and social pressure groups, reputation maintenance. However, there are correlations and crossovers among these triggers, and the classification is not clear. For example, if there are reports of social or environmental issues in the supply chain of an enterprise, customers will boycott their products, which will also affect the reputation.

### The Internal Factors

One of the main reasons why core companies are unwilling to be more active on sustainability issues is the lack of information, resources, and expertise in this area, leading to the poor ability of enterprises to achieve sustainable development (Zu and Kaynak, 2012; Bhakoo and Choi, 2013. Enterprise-level capabilities are a combination of various resources, such as material, human, and various corporate assets, including the “ability” of human resources (Eisenhardt and Martin, 2000). Gavronski et al. (2011) proposed that the cooperation between an enterprise and its supply chain partners embodies high-level capabilities. Over time, organizations face various changes and challenges from internal and external stakeholders, such as customers, suppliers, governments, competitors, etc. In this case, organizations must enhance capabilities and design strategies to cope with environmental changes (Freeman, 2010), which is consistent with the dynamic capability perspective (Teece et al., 1997). Enterprises must have the ability to use organizational processes to integrate, establish and re-allocate internal and external resources, design new value creation strategies to meet the sustainability requirements of stakeholders, reduce risks, and ensure their long-term performance (Teece et al., 1997). Resource availability also plays an important role in managing the sustainability in supply chain (Gong et al., 2019). The formation of social demand in the supply chain only depends on sufficient resources, but also on the ability of resource utilization (Mani et al., 2020). Compared with small and medium-sized enterprises, large enterprises can provide financial, human and technical resources to help their supply chain partners improve their sustainability performance (Vachon and Klassen, 2008; Wu et al., 2010). Large companies have more market force, so they are more influential among supply chain partners (Ayuso et al., 2013). In addition, Walters and James (2009) regard profitability and business efficiency as the driving force of core enterprises to promote and support supplier health and safety management.

Small and medium-sized enterprises are usually the suppliers of large enterprises, and more and more small and medium-sized suppliers are encouraged to take social responsibility and express these social responsibility requirements to their suppliers (Ayuso et al., 2013). However, sustainable supply chain management is challenging. Sub-suppliers may lack information and expertise and have weak relationships with core companies (Grimm et al., 2014; Wilhelm et al., 2016). Due to lack of resources and methods (Nawrocka et al., 2009), these small and medium-sized enterprises usually tend to communicate supply chain sustainability standards rather than implement control mechanisms (Touboulic and Walker, 2015). Although enterprises usually incorporate sustainability into their operations, they still don’t know how to extend sustainability to their supply chain partners through sustainable supply chain management, and drive and influence this process (Gong et al., 2019). Studies have shown that the ability used in environmental practices can help meet social needs, that is, the experience gained by enterprises in environmental management can be applied to social activities in supply chains (Van Hoof and Thiell, 2014).

The existing research literature shows that: (1) the existing research literature on the mechanism of the various influencing factors on the work safety management behavior of the core enterprise in supply chain mostly focuses on investigating the direct impact of each independent explanatory variable on the sustainable behavior of the enterprise supply chain, but the different influence paths of various variables are rarely accurately explored. (2) The existing literature shows some driving factors for the sustainable management of the core enterprise supply chain, and it is expounded from external factors such as stakeholder pressure and internal factors such as corporate capabilities. There are correlations and crossovers among external factors. The pressure exerted by stakeholders, the transmission from pressure to behavior, and the driving force of other internal factors (such as corporate management awareness) on corporate behavior are rarely explored. (3) There is limited literature on work safety management behaviors in supply chain of core enterprises. Although many variable categories studied in the literature (such as green supply chain management behavior, supply chain sustainable management behavior, supply chain social responsibility management behavior, etc.) are related to supply chain work safety management behavior. However, the connotation of these variables is not completely consistent with the work safety management behavior in supply chain. Supply chain work safety management is unique and should be independent of supply chain sustainability management, and it is worthy of attention.

Based on the relevant research results at home and abroad, the author studies the core enterprise supply chain work safety management behavior. The key factors and influence paths of enterprise supply chain safety management behaviors are explored in order to provide the theory and experience for the government to formulate effective guiding policies to promote the core enterprises to adopt supply chain work safety management behavior.

## Research Methods and Data Sources

At present, there is no mature variable category, measurement scale and theoretical hypothesis for core enterprise supply chain safety management behavior. In addition, according to field investigation, many enterprises have different views on supply chain work safety management behaviors, so it may not be effective to directly design undifferentiated structured questionnaires to conduct a large sample quantitative research on the public. Therefore, unstructured questionnaires (open-ended questionnaires) are used to interview relevant principals of representative core enterprises to collect first-hand information, and qualitative research is used to explore the supply chain safety management behavior of core enterprises more effectively. The interview subjects who may guide the development of the theory are selected through the theoretical sampling. Since qualitative research requires interviewees to understand the work safety management in supply chain, a core company in a supply chain with many suppliers is selected to conduct in-depth interviews on its purchasing managers, supply chain sustainability managers, EHS (environment, health and safety) managers, production managers or manager of supply chain technology department. All interviewees have worked in positions related to work safety management in supply chain for more than 5 years and are familiar with the process and system of cooperation between the enterprise and suppliers. Determine the number of samples based on theoretical saturation, that is, when new data do not produce new concepts and categories, the theoretical sampling process stops. Finally, 16 middle and senior managers from 12 core enterprises in China were interviewed. During the interview, after asking for permission, we recorded the content (the total recording length was 935 min). After the interview, we sorted out the recording materials (the interview transcript was 210,000 characters in total) and completed the interview records and memos. Table 1 shows the basic information of in-depth interview. The interview records of 8 enterprises were randomly selected for coding analysis and model construction, and theoretical saturation test was conducted for the interview records of the other 4 enterprises.

**TABLE 1:** Basic information of in-depth interview.

Enterprise	Business	The recording duration	Total characters recorded	Survey content	Usage
Enterprise 1	Aviation generator design and manufacture	90 min	25000 characters	Interviewed the director of sustainable development twice	Coding
Enterprise 2	Automobile R&D and manufacturing	50 min	8000 characters	Interviewed the supply chain technical manager once	Coding
Enterprise 3	Auto parts R&D and production	40 min	8000 characters	Interviewed EHS manager once	Coding
Enterprise 4	Medical product R&D and production	90 min	18000 characters	Interviewed EHS manager once	Coding
Enterprise 5	Passenger car R&D and manufacturing	90 min	22000 characters	Interviewed supplier development section chief once	Coding
Enterprise 6	Chip R&D and manufacturing	45 min	9000 characters	Interviewed the production manager once	Coding
Enterprise 7	Automobile R&D, production and sales	40 min	7000 characters	Interviewed the manager of supply chain management department once	Coding
Enterprise 8	Auto parts R&D and production	60 min	15000 characters	Interviewed EHS manager once	Coding
Enterprise 9	Drug R&D and manufacturing	100 min	26000 characters	Interviewed EHS manager and purchasing manager once	Testing
Enterprise 10	Decorative building materials production	60 min	10000 characters	Interviewed purchasing manager once	Testing
Enterprise 11	Diesel engine R&D and production	120 min	30000 characters	Interviewed purchasing manager and production manager once	Testing
Enterprise 12	Automotive sensor R&D and production	150 min	32000 characters	Interviewed general manager once, and interviewed purchasing manager once	Testing

**FIGURE 1:**
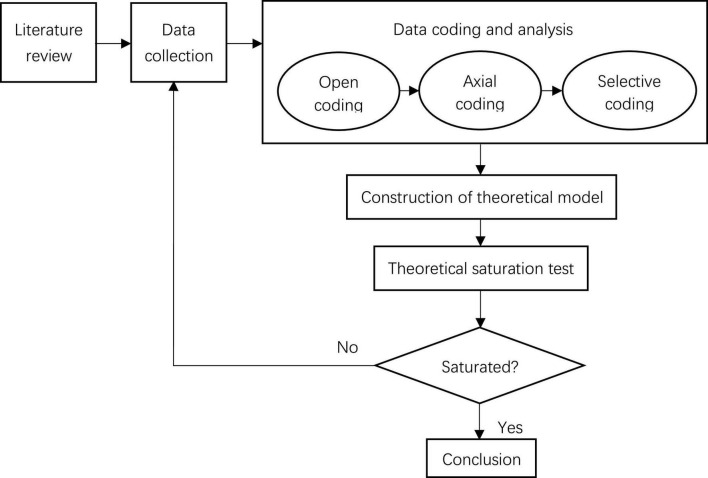
Research process of grounded theory.

Grounded Theory, the exploratory research technology, is mainly adopted in this study. Through three steps of open coding, axial coding and selective coding for text data, the theory of core enterprise supply chain work safety management behavior and its driving factors are constructed. The data are continuously compared to refine and revise theories until theoretical saturation is realized (that is, newly acquired data no longer contribute to theoretical construction). The grounded theory research process of this study is shown in Figure 1.

## Category Refinement and Model Construction

### Open Coding

Open coding is to code, label, and log the original interview data word by word to generate initial concepts and discover conceptual categories from the original data. In order to reduce the bias, opinion or influence of researchers, the respondents’ own words are used as labels to extract initial concepts. In this way, more than 600 original statements and corresponding initial concepts are obtained. Since there are many initial concepts and there is overlap among them, and category is the reclassification and combination of concepts, the initial concepts obtained are further categorized. When they are categorized, the initial concepts with low repetition frequency (less than 3 times) are eliminated, and the initial concepts with repetition frequency of more than 3 times are selected. In addition, individual inconsistent and irrelevant initial concepts that have nothing to do with the subject are eliminated. Table 2 shows the initial concepts and several categories obtained. In order to save space, three original data sentences and corresponding initial concepts are selected for each category.

**TABLE 2:** Open coding results.

Category	Source material	Initial concept
Regulatory pressure	“Chapter 7 of the “Medical equipment production quality management specification” has clear requirements for procurement, for example, when we purchase, screening supplier, supplier audit, and supplier control are clarified.”	Policy document requirements
	“State Drug Administration of the people’s Republic of China monitors us and audits our work, and it checks our key suppliers.”	Government Regulation
	“The concept of supply chain safety appeared for the second time around 2017, led by the Ministry of Industry and Information Technology of the People’s Republic of China, and it was called “Green Supply Chain.”	Idea transmission
Normative pressure	“Suppliers must first be able to comply with safety requirements. If something happens and it is reported by the media, they will say our company did it on purpose because the cost is cheaper.”	Media attention
	“To do business in this industry, one should comply with the industry’s regulations. This is an industry with higher barriers.	Industry specification
	“For the case of Apple, the toxic n-hexane was used in Apple’s supplier, which caused great negative social effects on Apple, as well as on the supplier.”	Social pressure
Cognitive pressure	“In the industry, my peers or my customers have already applied these supplier safety management specifications.”	Partner behavior
	“This is driven by the customers.”	Customer demand
	“Supplier safety management is conducted to reduce costs and make us more competitive.”	Competitive pressure
Responsibility sense	“We should co-exist and develop with suppliers, instead of producing by ourselves. Therefore, there is a high demand for supply chain sustainability.”	Concept of supply chain sustainability
	“At present, the transmission to suppliers is mainly based on the enterprise’s sense of responsibility and territorial awareness.”	Supply chain social responsibility
	“Generally, in large companies, the top-level design is pushed down with advanced vision, such as the US headquarters.”	Incorporated into the top-level design
Problem cognition	“In the automotive industry, once a supplier in supply chain is in trouble, our production and delivery will be affected.”	Affecting product delivery
	“So, supplier safety is as important as product quality, and their weight is very important. If the supplier is not safe, it will affect the product quality.”	Affecting product quality
	“For the medical industry, the cost of *ex post* control is (very large), and should I recall it? Should I suspend business for rectification after I was audited by various drug administrations and a finding was made? It is impossible. Therefore, most of supervision and policies are controlled in advance.”	Problem severity perception
Behavioral effect perception	“The role and weight of the enterprise in the closed loop of supply chain determines whether an enterprise can dominate or play role in the supply chain.”	Behavioral influence
	“I need to gain enough more voice in this industry, then I will release this information to my customers and suppliers for a virtuous circle. It makes me achieve a better position in the supply chain, so I can get goods with lower price.”	Behavior enhances industry status
	“The common growth and assistance for suppliers is to reduce costs and increase efficiency, in fact, for economic benefits.”	Economic value
Safety management strength	“When a company wants to conduct safety, in fact, it needs to develop a well understanding of safety, so that it can express a clearer and definite concept of safety to its industrial chain.”	Management knowledge
	“How do we keep up with the requirements of this regulation, and how do we find changes in regulations, and how do we pass these changes on to suppliers? The second is how to make the supplier know its importance.”	Ability to communicate safety specifications
	“For work safety problem, we use examples from our language to illustrate.”	Ability to express safety requirements
Safety coordination capability	“Emphasis on compliance with the law is a bit of significance, and they will be calmer to do things.”	Ability to coordinate conflict
	“How to help him and how to save the money. Conduct a return on investment calculation with the investor, and then we find a third-party financial company.”	Ability to utilize third-party service resources
	“Most of the money is still in the return on investment. You make some savings, but everybody can earn profit.”	Ability to share revenue
Safety evaluation	“We will regularly conduct on-site audits on suppliers and carry out some specific work.”	Regular audit
	“The supplier’s audit record, supplier’s evaluation report, and production record must be handwritten.”	Performance evaluation
	“If we find that there are major risks in suppliers, first, it is fined; second, reduce order allocation; third, if it is not improved, we may give him a red item in the annual audit, and may suspend its new project development.”	Punishment
Safety cooperation	“There was mechanical crushing of suppliers that almost killed workers. In this case, we will provide technical assistance to suppliers.”	Technical guidance
	“If some small suppliers are met with difficulties, Korean or Japanese companies will provide financial support.”	Financial assistance
	“The suppliers may have poor performance in work safety, and we will send our work safety administrator to provide training.”	Management guidance

*The words in parentheses at the end of each sentence represent the initial concept obtained by encoding the original sentence.*

### Axial Coding

The purpose of axial coding is to discover the logical relationships among categories, and to develop the main category and the corresponding sub-categories. According to the conceptual and logical relationship between different categories, they are classified into four main categories. Based on the relationship among different categories in concept level and logical level, four main categories are concluded. Table 3 shows the main categories and their corresponding open coding categories.

**TABLE 3:** The main categories formed by the axial coding.

Main categories	Corresponding categories	Relationship connotation
Institutional pressure	Regulatory pressure	Requirements from laws and regulations, government supervision and industry standards will affect the institutional pressure of core enterprises to implement supply chain safety management
	Normative pressure	The supervision and attention from the public, the media and the community will affect the institutional pressure of core enterprises to implement supply chain work safety management
	Cognitive pressure	The practices of partners, the behaviors of competitors, and the effects of competitors’ management behaviors will affect the institutional pressure of core companies to implement supply chain safety management
Behavioral awareness	Responsibility sense	Whether the enterprise has a sense of social responsibility in the supply chain, the sustainable development of the supply chain, the sense of social responsibility, the support of senior management, the enterprise’s attention on supply chain safety, and whether it is incorporated in the top-level design of the company’s supply chain management will affect the behavioral awareness of core enterprise’s implementation of supply chain work safety management
	Problem cognition	The accidents of supply chain work safety accidents affect the enterprise’s product delivery, product quality, and brand reputation. Awareness of the seriousness of these problems will affect the core enterprises’ awareness of the behavior of supply chain work safety management
	Behavioral effect cognition	The influence of enterprises adopting supply chain work safety management behaviors; customer recognition, media public recognition, and government recognition brought by management behavior; the change of management behavior on the enterprise’s industry status affects the behavioral awareness of work safety management in the supply chain of core companies
Behavioral capability	Safety management strength	The understanding of knowledge of enterprise on work safety management and the ability to understand work safety regulations and express safety requirements and communicate specifications to suppliers affect the behavioral capability of core enterprise supply chain work safety management
	Safety coordination capability	The ability to coordinate conflicts when cooperating with upstream and downstream enterprises in the supply chain, and the ability to share safety risks and benefits with upstream and downstream enterprises affect the core enterprise’s ability to conduct work safety management in supply chain
Management behavior	Safety evaluation	Formulating supplier safety supervision procedures, evaluating the supplier work safety performance, and providing rewards and punishments based on the evaluation results constitutes the core enterprise supply chain work safety management behavior
	Safety cooperation	The enterprise assisting suppliers in obtaining work safety certification, providing suppliers with work safety technical support, funding channels, and management guidance form the core enterprise supply chain work safety management behavior

### Selective Coding

Selective coding is to dig out the connection among the main categories, and describe behavioral phenomenon and contextual conditions in a “story line” manner. After the “story line” is finished, a new substantive theoretical framework can be developed. The typical relationship structure of main categories is shown in Table 4.

**TABLE 4:** Typical relationship structure of the main categories.

Typical relationship structure	The connotation of relationship structure
pressure → behavior	Institutional pressure is the external driving factor of the core enterprise supply chain work safety management behavior, and it can directly affect the core enterprise supply chain work safety management behavior
awareness → behavior	Behavioral awareness is the internal driving factor of the core enterprise supply chain work safety management behavior, and it directly affects the core enterprise work safety management behavior in supply chain
capability → behavior	Behavioral capability is the internal driving factor of the core enterprise supply chain work safety management behavior, and it directly affects the supply chain work safety management behavior of core enterprises
pressure → awareness → behavior	Institutional pressure affects management behavior by influencing the behavioral awareness of supply chain work safety management of core enterprises
pressure → capability → behavior	Institutional pressure affects management behaviors by influencing the core enterprise’s supply chain work safety management ability
awareness ↓ pressure—behavior	As an internal condition, behavioral capability affects the relationship intensity and direction between institutional pressure and management behavior
capability ↓ pressure—behavior	As an internal condition, behavioral capability affects the relationship intensity and direction between behavioral awareness and managerial behavior

**FIGURE 2:**
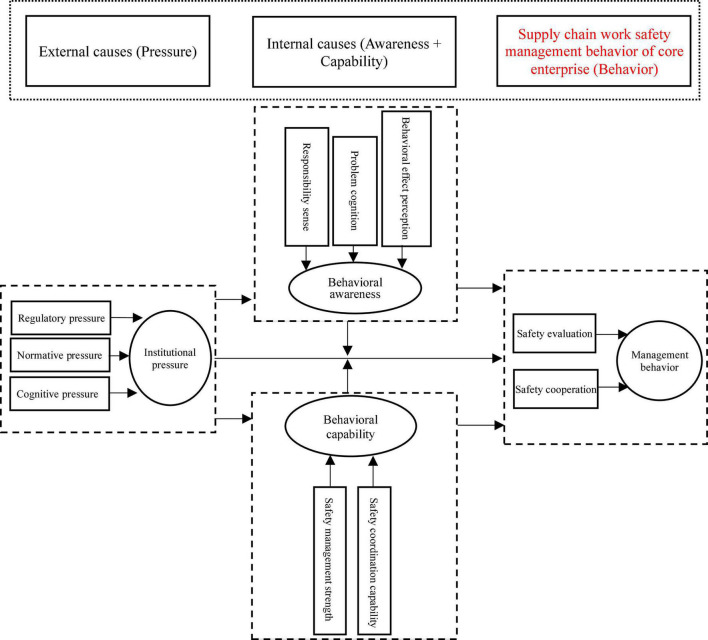
Driving mechanism model.

“The driving factors and driving mechanism of the core enterprise supply chain work safety management behavior” is defined as the core category. The “story line” on the core category can be summarized as: institutional pressure, behavioral awareness, and behavioral capability have a significant impact on the main category of core enterprise supply chain work safety management behavior; institutional pressure is an external driving factor, and behavioral awareness and behavioral capability are internal driving factors; on the one hand, institutional pressure directly affects management behavior, on the other hand, it indirectly affects management behavior through behavioral awareness and behavioral capability; behavioral awareness and behavioral capability directly affect management behavior; behavioral awareness and behavioral capability can adjust the connection between pressure and management behavior. Based on the “story line,” the driving mechanism model of the core enterprise supply chain work safety management behavior is constructed and developed, as shown in Figure 2.

### Theoretical Saturation Test

The other 1/3 of the interview records are applied for the theoretical saturation test. The results show that the categories in the model have been developed completely. For the three main categories (institutional pressure, behavioral awareness, and behavioral capability) affecting the supply chain work safety management behavior of the core enterprise, there are no new important categories and relationships. No new constituent factors are found in the four main categories, including management behavior. Hence, the model is theoretically saturated.

## Explanation of the Driving Mechanism Model

Through the previous analysis, it is found that the “external cause-internal cause-behavior model” can effectively explain the mechanism of the supply chain work safety management behavior of core enterprises. Specifically, the power elements of the supply chain work safety management behavior of the core enterprises can be summarized into three main categories: institutional pressure, behavioral awareness and behavioral capability, which basically follow the general dynamic structure law of the development of things. However, their mechanism on the supply chain work safety management behavior of the core enterprises (that is, the way and path they affect the management behavior) is not completely consistent. The details are explained below.

### The Direct Effect of Institutional Pressure

Institutional pressure (determined by the regulatory pressure, normative pressure, and cognitive pressure) is the external environmental factor of the enterprise that generates the motivation for work safety management in the supply chain. On the one hand, institutional pressure directly affects the supply chain work safety management behavior of the core enterprises. On the other hand, it also affects management behavior by acting on behavioral awareness and behavioral capability, respectively. It is the strengthening power for enterprises to conduct supply chain work safety management behavior. The source and intensity of institutional pressure will affect the positive relationship between pressure and behavior. (1) The source of institutional pressure will affect the positive relationship between pressure and behavior. When the pressure comes more from the government guidance, the management standards set by industry associations, the assistance of non-governmental organizations, and the excellent practices of partners, such source pressure will have a more significant positive effect on management behavior than pure moral preaching. For example, one manager mentioned “morality, I am very disgusted by such words. Why when we talk about work safety management in the supply chain, the enterprise social responsibility comes in? Enterprise social responsibility is all empty talk.” (2) The intensity of institutional pressure will affect the positive relationship between pressure and behavior. When the institutional pressure is weak, the core enterprises hardly adopt the work safety management behavior in supply chain. At present, there are no relevant laws and regulations in China that clearly state that enterprises should be responsible for the work safety of their suppliers, so some enterprises do not pay attention to the work safety management in supply chain. It can be found from some of the views of the interviewees. For example, “The government has no requirements, and there is no requirement for safety management in supply chain. It focuses on quality management and quality assurance.” It can be seen that when the institutional pressure reaches a certain critical point, as the institutional pressure increases, the motivation for enterprises to adopt supply chain work safety management behaviors is significantly enhanced. In addition to the source and intensity of institutional pressure, the way in which it acts will also affect its positive effect on management behavior. This will be explained in detail below.

### The Mediating Role of Internal Motivations

Most previous studies focused on the direct impact of external factors such as institutional factors or stakeholder pressure on supply chain management behavior. Through in-depth interviews and qualitative research in this paper, it is found that the significance of internal factors in enterprise on the supply chain work safety management behavior of the core enterprises cannot be ignored. The path of external factors on management behavior is explored. The internal motivations of the supply chain work safety management behavior of enterprises include behavioral awareness (the subjective initiative factor of the enterprise) and the behavioral capability (the objective regularity of the enterprise). Behavioral awareness is composed of responsibility sense, problem cognition and behavioral effect perception. The behavioral capability is composed of safety management strength and safety coordination capability. On the one hand, the internal motivation plays an intermediary role in the relationship between institutional pressure and management behavior, that is, institutional pressure affects management behavior indirectly through the internal motivation. Specifically, institutional pressure promotes the management behavior of the enterprise by enhancing the awareness or capability of the supply chain work safety management behavior. For example, on the one hand, pressure from the government or society can stimulate enterprises to meet external legal requirements. On the other hand, the guidance and assistance from the government or society can enhance the internal and external strength of enterprises to participate in the work safety management in supply chain. First, the production and development of an enterprise requires its interaction with stakeholders, and gaining the recognition of stakeholders is the prerequisite for interaction. The external environment exerts institutional pressure on enterprises. In order to gain recognition, enterprises need to meet the demands of stakeholders. Therefore, the awareness of work safety management behavior in supply chain and management willingness are generated. Second, the core enterprises supply chain work safety management capability is a dynamic capability, in addition to the enterprise capabilities and foundation, its cultivation and promotion requires external resources and social capital. Obtaining resources across organizational boundaries can expand the corporate knowledge pool and support the cultivation of corporate behavioral capabilities through resource supply. The external institutional environment can provide enterprises with a resource basis for work safety management in supply chain, and empower them. Enterprises with strong behavioral capabilities can seize opportunities through the resource integration to adopt supply chain work safety management behaviors. The external institutional pressure influences management behavior indirectly by cultivating the behavioral awareness and behavioral capability of core enterprises. Therefore, behavioral awareness and behavioral capability, as effective intermediary between institutional pressure and management behavior, have a pulling effect on the supply chain work safety management behavior of enterprises.

### The Moderating Effect of Internal Motivation

On the other hand, internal motivations act by influencing the relationship strength and direction between institutional pressure and management behavior, so they are moderating variables. The moderating effect of behavioral awareness is affected by the source and intensity of the awareness. When the behavioral awareness is strong or comes from the factors that affect the vital interests of the enterprise, such as brand maintenance, product quality assurance and supply chain risk control, that is, when enterprises perceive that it is profitable to implement supply chain work safety management, the moderating effect of behavior awareness is more significant. For example, some interviewees said, “So it is conducted based on interests. Since the enterprise is profitable and can grow rapidly, and it can reflect social responsibility, why not do it”? Conversely, when behavioral awareness comes from the ethical requirements of social responsibility and taking management behaviors does not benefit the actual operation of the enterprise, the moderating effect of behavior awareness is weak. For example, some managers said, “in our industry, there is little demand.” “In fact, I think this kind of risk is acceptable, and there is no need for supplier work safety management.”

The moderating effect of behavioral capability is affected by the characteristics of capability, which include strength and structure. When an enterprise has strong behavioral capabilities or comprehensive capabilities, that is, the enterprise not only has a high level of work safety management, but also has the corresponding supply chain safety management capabilities, its moderating effect is more significant; conversely, the moderating effect of behavioral capability is small. For example, one interviewee said, “The work safety management capability in supply chain depends on an enterprise’s background, foundation and people inside. It is a comprehensive evaluation. The more successful of the enterprise is, the talents it chooses should be professionals with certain qualities and planning capabilities.”

When the institutional pressure is weak and the moderating effect of internal motivations is relatively strong, the supply chain work safety management behavior of core enterprises is more affected by internal motivations. For example, one manager said, “If the government’s financial incentive is only a one-time policy, it is only the icing on the cake for the enterprise. Promoting enterprises to implement supply chain safety management requires the comprehensive strength of enterprises themselves.” Therefore, when the institutional pressure is weak, enterprises do not take management actions because of low input and output. Conversely, when the institutional pressure is high, the moderating effect of internal motivations is relatively weak.

## Results and Discussion

### Results

In the current global economy, enterprises are gradually relying on outsourcing some business activities and processes, and enterprises and their suppliers are in a specific network, that is, a supply chain network. As the center of the supply chain, the core enterprises regulate and govern the supply chain, maintain direct contact with customers, and design and provide products and service (Seuring and Müller, 2008). The fulfillment of enterprise functions depends on the level of supply chain. This trend of outsourcing and the increasing importance of supply chain influence the working conditions, health and safety of supplier workers. Therefore, this paper studies the internal and external factors that influence core enterprises to adopt the behavior of supply chain work safety management to put forward policy suggestions for the government to guide core enterprises to participate in supply chain work safety governance, improve the work safety level of small and medium-sized suppliers and the sustainable development of the whole supply chain.

This study shows that institutional pressure, behavioral awareness and behavioral capability have a significant impact on the core enterprise supply chain work safety management behavior. Among them, institutional pressure is the external motivation for enterprises to adopt management behavior, and behavioral awareness and behavioral capability are the internal motivations. On this basis, this paper explores and constructs the mechanism model of the above three main categories on enterprise supply chain work safety management behavior. The main findings include: (1) the supply chain work safety management behavior of the core enterprises is not only affected by external institutional pressure, but also by the internal behavioral awareness and behavioral capability. (2) The formation mechanism and constituent factors of institutional pressure, behavioral awareness, behavioral capability, and supply chain work safety management behavior are explored. For example, the constituent factors of behavioral awareness include responsibility sense, problem cognition and behavioral effect perception; the constituent factors of behavioral capability include safety management strength and safety coordination capability. The sub-categories of problem cognition, behavioral effect perception and behavioral capability have not been emphasized and categorized in previous literature. (3) The influence mechanism of institutional pressure on behavior and the mediation and moderation of internal motivations on the relationship between pressure and behavior, especially the source and intensity of institutional pressure affect the positive relationship between pressure and behavior; the institutional pressure affects the enterprise management behavior through behavioral awareness or behavioral capability; the moderating effect of behavioral awareness is affected by the source and intensity of awareness; the moderating effect of behavioral capability is affected by the strength and structure of the capability; the moderating effect of internal motivations and institutional pressure are in a trade-off relationship. These findings inspire for the government guidance measures proposed in this paper, that is, simply increasing pressure cannot significantly drives enterprises to participate in work safety management in supply chain, and it is necessary to pay attention to the source and composition of external institutional pressure, and clarify whether external pressure promotes behavioral awareness and behavioral capability within the enterprise.

### Contributions

#### Theoretical Contributions

Core enterprises to adopt effective work safety management behaviors in supply chain is conducive to reduce supply chain safety risks, and is a beneficial supplement to the government safety supervision. That is, the market mechanism is used to improve the work safety level of small and medium-sized suppliers. However, existing studies lack an in-depth exploration of the work safety management behaviors in supply chain of core enterprises. At the same time, existing studies on the drivers of supply chain sustainability management integrate work safety into the sustainability management, lacking consideration of the uniqueness of work safety management in supply chain. Hence, through an in-depth analysis of typical enterprises’ supply chain work safety management practices, the author proposes a model of core enterprises’ supply chain work safety management behavior driving mechanism around the uniqueness of supply chain work safety management, and clarifies that core enterprises’ adoption of supply chain work safety management behavior is influenced by external institutional pressure, enterprise behavior awareness and enterprise specific behavior capability. Unlike existing studies focusing on examining the direct impact of each independent variable (considering external stakeholder pressure factors or enterprises’ internal capacity factors) on enterprises’ supply chain safety sustainability behavior, the author specially studies the supply chain safety management independently of sustainable supply chain management. Second, it is found that internal factors (including supply chain safety responsibility awareness, perception of supply chain safety issues, perception of effectiveness of safety management behaviors in supply chain, corporate safety management strength and supply chain safety coordination capability) are important driving forces of work safety management behaviors in supply chain that cannot be ignored. Finally, the author does not stop at listing the internal and external drivers that influence the adoption of supply chain safety management behaviors by core enterprises, but further focuses on the transmission mechanisms of internal and external factors on enterprises’ work safety management behaviors in supply chain. Safety management is a unique and important issue that is fundamental to the development of corporate production and it should be emphasized in supply chain management of core companies. This study can draw attention to the uniqueness of the work safety management in the study of behavior-driven mechanisms of supply chain sustainability management.

#### Practical Contributions

The participation of core enterprises in supply chain work safety management is an important complement to government work safety controls. Although existing research on supply chain sustainability management gives some ideas and measures to drive enterprises to adopt sustainable behaviors, these measures lack the relevance to address the lack of motivation of enterprises to adopt work safety management behaviors in supply chain. The model of core enterprises’ supply chain work safety management behavior driving mechanism in this paper can be used to provide targeted policy ideas and implementation paths for the government to develop effective control policies to guide core enterprises to adopt effective supply chain work safety management behaviors. The details are described below.

(1) Create an institutional environment to enhance behavioral awareness:

Firstly, motivate the supply chain sustainable development concepts and supply chain social responsibility of core enterprises through a variety of methods. Some core enterprises lack the concept of sustainable development in supply chain, and fail to realize that competition among enterprises has evolved into competition among supply chains. Therefore, the government and society should spread management concepts, create an environment of public opinion, stimulate the management awareness of core enterprises, guide more enterprises to participate in supply chain work safety management, and create a safe and healthy supply chain network. Secondly, improve the awareness of work safety management in supply chain. Some core enterprise managers lack awareness of the work safety in supply chain. Lastly, improve core enterprises’ perception of the effect of supply chain work safety management behavior. Some core enterprises only regard supply chain work safety management as an extra cost expenditure, with fixed thoughts, and do not take the initiative to explore new management innovation mode to seek the economic value of supply chain work safety management. From the perspective of the profit-seeking nature of capital, the economic benefits of social responsibility behaviors are the internal driving force for enterprises to fulfill their social responsibilities. From the perspective of strategic social responsibility thinking, Porter and Kramer (2006) pointed out that competitive advantage is the result of the integration between the social responsibility strategy and the internal and external environment of enterprises (the government creates the environment). If enterprises take the initiative to incorporate social responsibility into their strategy, fulfilling social responsibility can become a source of opportunity, innovation and competitive advantage (Nawrocka et al., 2009). Strategic management of social responsibility can transform the undertaking of social responsibility from cost to enterprise resources and capability that can create value to form competitive advantage. On the one hand, the government can promote the influence of enterprises in the industry by advocating enterprises to participate in the formulation of industry norms. On the other hand, all sectors of society need to advocate enterprises to operate in a responsible and sustainable way, make positive contributions to the safe and healthy development of supply chain, and bring more opportunities for innovation, stronger partnership and more sustainable growth for enterprises themselves.

(2) Help companies improve their behavioral capabilities:

The way for sustainable development of the supply chain lies in improving management capabilities. While achieving its own sustainable development, it will drive the sound development of the entire supply chain, prompt upstream suppliers to further regulate their production behavior and production methods, and attract potential suppliers to actively achieve work safety. Standardizing supply chain work safety management requires not only sufficient resources but also the ability to use resources effectively (Mani et al., 2020). The quality level of suppliers is uneven, as is the core enterprises. In order to improve the behavioral capabilities of work safety management in supply chain, first, it is necessary to improve the safety management strength of core enterprise, including safety management knowledge, technical strength, and human resources. Only when an enterprise has its own safety management strength and a good safety performance can it spread safety concepts and management methods to the upstream and downstream in supply chain. Second, enhance the safety communication and coordination capabilities of core companies in the supply chain. Establish information exchange media and platforms, organize core enterprise supply chain security management exchange conferences, and encourage enterprises to exchange experiences and learn from each other’s excellent management practices. When the core enterprise cooperates with the upstream and downstream in supply chain, learn how to coordinate the conflicts between the work safety requirements and the production and operation of the supplier enterprise, and how to share the safety risks and the safety benefits with the upstream and downstream enterprises.

Finally, interactive learning should be advocated. Enterprises in the supply chain network should learn from each other’s excellent management experience. The government and all sectors of society should create an institutional environment. Through the stakeholder network, explore and publicize the management model, advocate interactive learning and set up industry benchmark, and enhance the management capability of core enterprises. The government and all sectors of society provide more resources and recruit more relevant parties, such as NGOs and work safety service agencies, to assist core enterprises in supply chain work safety management. The unified understanding and collective action of all supply chain members and the dialog and information sharing among supply chain members are conducive to forming consensus and synergy, maximizing the advantages of supply chain enterprises and jointly creating a safe and healthy supply chain.

### Limitations and Future Work

The limitations and future work of this study are as follows. Since the supply chain work safety management behavior and its driving factors model are proposed based on the exploratory study, the reliability, validity and popularization have not been tested by large-sample statistics, in the future, it is necessary to conceptualize the variable categories involved in the model and develop a measurement scale, and a large-scale questionnaire survey can be used to test the exact relationship among the variables in the model. Furthermore, when the government and all walks of life are formulating policies and systems that guide more enterprises to participate in work safety management in supply chain, what is the mechanism and effect of a specific policy system for promoting the enterprise management behavior, and how to coordinate different policies to maximize the integration effect can be determined through setting up scenario experiments and simulation by using computer.

## Data Availability Statement

The original contributions presented in the study are included in the article/supplementary material, further inquiries can be directed to the corresponding author/s.

## Author Contributions

QM provided the financial support and designed the manuscript. QZ and SL carried out the formal analysis. QZ and JZ wrote the original draft of the manuscript. QW performed the methodology. All authors contributed to the article and approved the submitted version.

## Conflict of Interest

The authors declare that the research was conducted in the absence of any commercial or financial relationships that could be construed as a potential conflict of interest.

## Publisher’s Note

All claims expressed in this article are solely those of the authors and do not necessarily represent those of their affiliated organizations, or those of the publisher, the editors and the reviewers. Any product that may be evaluated in this article, or claim that may be made by its manufacturer, is not guaranteed or endorsed by the publisher.
